# Signal-piloted processing and machine learning based efficient power quality disturbances recognition

**DOI:** 10.1371/journal.pone.0252104

**Published:** 2021-05-28

**Authors:** Saeed Mian Qaisar

**Affiliations:** 1 Electrical and Computer Engineering Department, Effat University, Jeddah, Saudi Arabia; 2 Communication & Signal Processing Lab, Energy & Technology Research Centre, Effat University, Jeddah, Saudi Arabia; Vellore Institute of Technology: VIT University, INDIA

## Abstract

Significant losses can occur for various smart grid stake holders due to the Power Quality Disturbances (PQDs). Therefore, it is necessary to correctly recognize and timely mitigate the PQDs. In this context, an emerging trend is the development of machine learning assisted PQDs management. Based on the conventional processing theory, the existing PQDs identification is time-invariant. It can result in a huge amount of unnecessary information being collected, processed, and transmitted. Consequently, needless processing activities, power consumption and latency can occur. In this paper, a novel combination of signal-piloted acquisition, adaptive-rate segmentation and time-domain features extraction with machine learning tools is suggested. The signal-piloted acquisition and processing brings real-time compression. Therefore, a remarkable reduction can be secured in the data storage, processing and transmission requirement towards the post classifier. Additionally, a reduced computational cost and latency of classifier is promised. The classification is accomplished by using robust machine learning algorithms. A comparison is made among the k-Nearest Neighbor, Naïve Bayes, Artificial Neural Network and Support Vector Machine. Multiple metrics are used to test the success of classification. It permits to avoid any biasness of findings. The applicability of the suggested approach is studied for automated recognition of the power signal’s major voltage and transient disturbances. Results show that the system attains a 6.75-fold reduction in the collected information and the processing load and secures the 98.05% accuracy of classification.

## 1. Introduction

### 1.1. Background and problem statement

Several problems are raised in the conventional and smart grids due to the PQDs [1]. Such irregular events can significantly affect the output of power networks. The PQ events mainly influence one or more attributes of the supply such as frequency and voltage [2]. They can appear after an accidental fuse of circuit breaker, problems with capacitor switching, use of nonlinear loads, electrical equipment abnormal functioning and overheating of transformers [3, 4].

The power consumption is globally increasing and an increased power generation by using conventional approaches could harm the environment. Therefore, in the aim of attaining the green supply, the trend is to integrate renewable energy sources in smart grids. However, integration of various energy sources is challenging and also originate the PQDs [5, 6].

The economy of any society can suffer from failures which occur due to the PQDs. It is particularly unbearable for sensitive facilities such as hospitals, data centres and nuclear reactors. Therefore, such customers are inclined to install the automated on-site PQDs recognition and mitigation equipment [5]. Such systems use the smart sensors, controllers and actuators for PQDs automated management. The recent advancements in the domain of machine learning have evolved a variety of automated solutions [7–14]. The same trend is followed by the automated PQDs mitigation techniques [15–17]. The PQDs identification should be carried out with precision and minimum possible latency. A timely assessment of PQDs permits to protect the critical and expensive power generation and distribution equipments and loads connected to the network. This could prevent the massive economical losses [4, 18].

PQDs are of non-stationary and sporadic nature. The monitoring mechanism characterizes these PQDs, their corresponding instance of occurrence and location. The findings can be used to pilot the appropriate actuators in order to mitigate the recognized PQDs in a quasi-real-time manner. To effectively mitigate PQDs, an uninterrupted monitoring of the power signals must be performed. It is a tiresome job and cannot be performed manually. Moreover, due to fatigue and lack of concentration mistakes can be made by the onsite power engineers. In this context, for the recognition and mitigation of PQDs, automatic approaches have been suggested [15, 16, 19–23].

### 1.2. Related works

In any automatic PQDs identification scheme, the power signals mainly goes through three steps namely conditioning, features extraction and classification [24, 25]. In [19] Borges et al. have used the time-domain and frequency-domain methods for the statistical features extraction. Onward, these extracted features are processed by the Artificial Neural Network (ANN) classifier for the PQDs identification. In [15] Hussain et al. have used the singular spectrum analysis and Curvelet transform methods for the features extraction. Onward, these extracted features are processed by the deep Convolutional Neural Network (CNN) classifier for the PQDs identification. In [20] Agüera-Pérez et al. have used the higher-order statistical analysis method for the features extraction. The categorization is achieved by using a higher-order statistical estimator. In [16] Manikandan et al. have used the sparse signal decomposition with hybrid dictionary for the features extraction. The categorization is achieved by using the hierarchical decision tree algorithm. In [21] Decanini et al. have used the discrete wavelet transform and entropy norm for the features extraction. The categorization is achieved by using the ANN. In [22] Singh et al. have used the non-dominated sorting genetic algorithm based on S-transform and time-time transform for the features extraction. Decision Tree (DT) is used for the classification purpose. In [23] Ferreira et al. have used the Independent Component Analysis (ICA) for the features extraction. Onward, the categorization is achieved by using the Multi Layer Perceptron (MLP).

### 1.3. Research gap and contribution

In the aim of achieving a precise identification and mitigation of the power quality issues, a fine grained power signal recording and processing is necessary [26]. Almost all existing automatic power signal processing systems operate at fixed-rate regardless of the rate of information in the incoming signal. It can cause a useless processing, storage and transmission of information [26]. In this framework, compressed sensing and analog–to–information conversion solutions have been devised [27].

In continuation of works presented in [24, 28, 29], this research enhances the existing fix-rate automated PQ events identifiers. Shortfalls of the classical fixed-rate power signal processing systems are compensated by using the signal-piloted power signals recording and analysis. It records the necessary details of the intended power signal while noticeably diminishing the amount of collected information. It brings a remarkable real-time compression along a computational load diminishing of the suggested adaptive-rate PQ events identifiers in comparison with the fix-rate counterparts [15, 16, 19–23]. The following are the key contributions of this paper.

- Suggesting an efficient and precise solution for major power voltage and transient disturbances recognition. It processes the power signal in an efficient adaptive-rate manner and extracts the classifiable features directly in time-domain without using any computationally complex transformation based approach.
- Intelligently combining the signal-piloted Analog to Digital Converters (SPADCs), Activity Selection Algorithm (ASA), time-domain features extraction and machine learning algorithms for an efficient classification of PQ events:
  - Power signals are acquired at adaptive-rates by using the SPADCs.
  - The segmentation is achieved by using the MASA
  - A novel features extractor directly analyzes each segment in time-domain to determine its discriminating attributes.
  - The PQDs categorization is realized by using the forehand time-domain extracted features by using robust machine learning algorithms.

### 1.4. Paper organization

The proposed signal-piloted and adaptive-rate power signal processing scheme is described in Section 2. The power quality disturbances model, novel time-domain features extraction and robust machine learning based classification algorithms are also described in Section 2. Moreover, Section 2 also presents the system performance evaluation measures. Section 3 presents an experimental evaluation of the devised method. Section 4 discusses the main findings and features of the suggested approach. It also presents a performance comparison with contemporary PQDs classification methods. Finally the conclusion is made in Section 5.

## 2. Materials and methods

**Fig 1:**
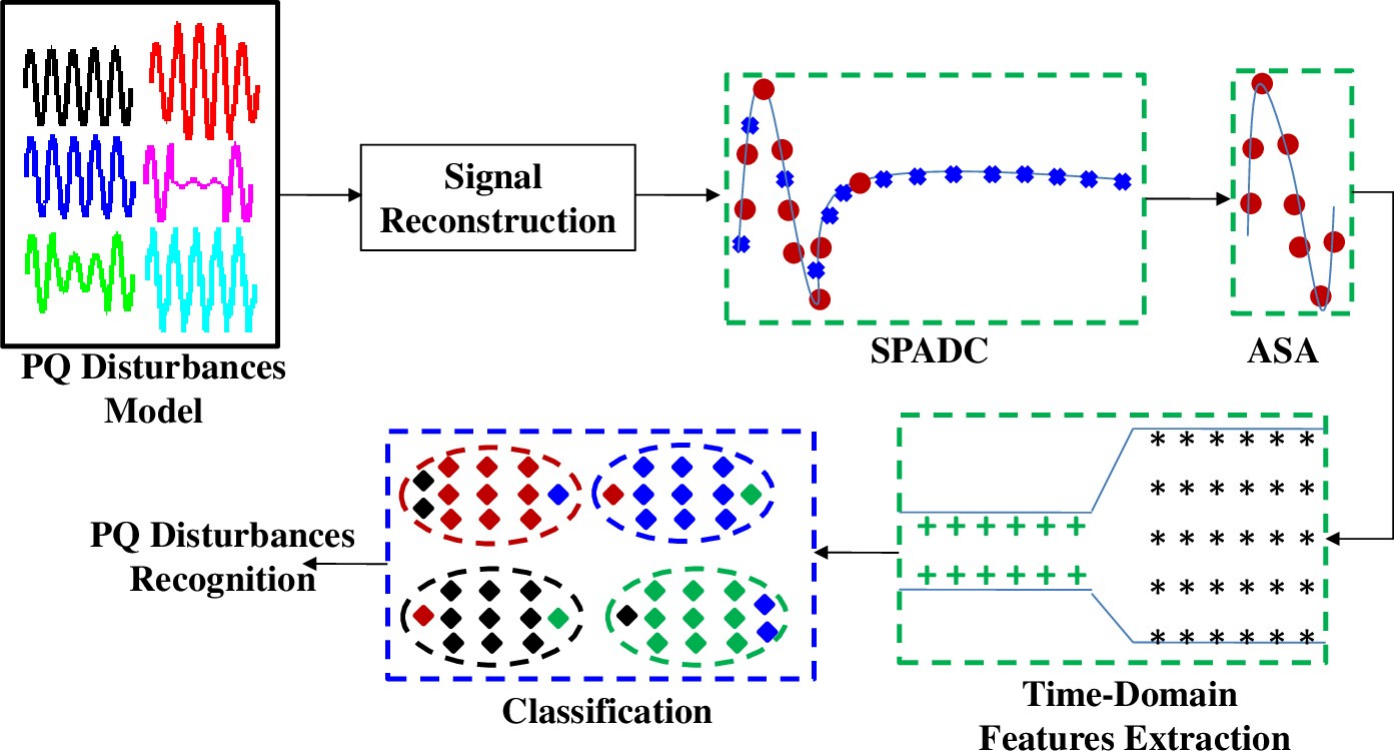
The suggested system block diagram.

The different stages of proposed system are displayed in Fig 1. Modules enclosed by the solid black colour line ‘^______^’ are modelling scenarios for testing the suggested solution. Modules enclosed by the dashed green colour line ‘**- - -**’represent stages, proposed to be embedded in the frontend processing chain. The dashed blue colour line ‘**- - -**’encloses the classification stage, which could be realized via a cloud or server based application. Implementing the optimized processing modules in frontend processor and keeping the classification operation on the cloud allows realizing an effective and configurable solution [30].

### 2.1. Power Quality Disturbances (PQDs) model

The collection of PQDs is not straightforward. Therefore, a common tendency is to generate the real-like signals by utilizing the mathematical models [2, 31]. The synthetic PQDs have been used in previous studies for the evaluation of proposed features extraction and recognition approaches [15–17, 20–22]. In this study four major categories of power signals namely pure signal, sag, interruption and swell are generated by using MATLAB R2019b [32]. The employed PQDs generation model is based on the IEEE recommended standard for PQDs and it generates PQDs waveforms in a random fashion [33, 34]. Concretely, the voltages and occurrence timings, of intended PQDs, are randomly changed. It is performed within the limits of their corresponding bounds, presented in [34]. In order to approach the realistic case, random noise is added in these signals in order to attain a Signal to Noise Ratio (SNR) of 40 dB [19].

A description of these power signals is provided in the following. For equal representation, 200 instances are considered from each category. It results in 800 instances in total. Each instance is produced for a half-second period, equal to the 25 cycles of pure power signal of frequency 50Hz. By following the process, presented in [19], it is sampled at a rate of 6.4 kHz. Each instance is composed of 3200 samples. It results in the dataset dimensionality of 800x3200.

#### 2.1.1. The Pure Signal (PUS)

It is the faultless power signal which represents a desired supply. Eq (1) introduces this phenomenon mathematically [33, 34]. Where, *A* and *f* are respectively the power signal amplitude and frequency. The f = 50 Hz is selected [33, 34]. *t*_*n*_ and *φ* are the instants of sampling and phase.

$$y1(t_{n})=A\;sin(2\pi f\;t_{n}-\varphi ).$$ (1)

#### 2.1.2. The sag

It causes the Root Mean Square (RMS) voltage to decrease briefly between 0.1–0.9 per unit (pu). It is a PQD that might last between half cycle to 1-minute [33, 34]. Sags can occur due to momentary short circuits or by turning on heavy loads such as big motors. It can also be caused by overloading transformers or due to the deployment of undersized conductors. Eq (2) introduces this phenomenon mathematically. *t*_*n*_ and *φ* are the instants of sampling and phase.

$$y2(t_{n})=A\;(1-\alpha (u(t_{n}-t_{1})-u(t_{n}-t_{2})))sin(2\pi ft_{n}-\varphi ).$$ (2)

In Eq (2), 0.1 ≤ *α* ≤0.9, *T* ≤ *t*_2_ –*t*_1_ ≤ 49*T* and $T=\frac{1}{f=50}$ Sec.

#### 2.1.3. The Interruption (Intr.)

It takes place due to defects in the power grid, equipment malfunction and controller shortfalls [31]. This causes loss of supply voltage for a limited period, usually less than a minute. It temporarily reduces the RMS voltage supply below 0.1 pu [33, 34]. Eq (3) introduces this phenomenon mathematically. Where, *t*_*n*_ and *φ* are the instants of sampling and phase.

$$y3(t_{n})=A\;(1-\rho (u(t_{n}-t_{1})-u(t_{n}-t_{2})))sin(2\pi ft_{n}-\varphi ).$$ (3)

In Eq (3), 0.9 ≤ *ρ* ≤ 1, *T* ≤ *t*_2_ –*t*_1_ ≤ 49*T* and $T=\frac{1}{f=50}$ Sec.

#### 2.1.4. The swell

It can be originated by turning off a heavy load or by energizing a large network of condensers [31]. It causes a momentarily rise in the power line voltage. When renewable energy sources such as solar panels are linked to the network, they can also originate swells. It causes the RMS voltage to increase briefly between 1.1–1.8 pu. It is a PQD that might last between half cycle to 1-minute. Eq (4) introduces this phenomenon mathematically [33, 34]. Where, *t*_*n*_ and *φ* are the instants of sampling and phase.

$${\text{y}4(\text{t}}_{\text{n}})=\text{A}\;{(1+\beta (\text{u}(\text{t}}_{\text{n}}{-\text{t}}_{1}{)-\text{u}(\text{t}}_{\text{n}}{-\text{t}}_{\text{2}}{)))\text{sin}(2\pi \text{ft}}_{\text{n}}-\emptyset ).$$ (4)

In Eq (4), 0.1 ≤ *β* ≤ 0.8, *T* ≤ *t*_2_ –*t*_1_ ≤ 9*T* and $T=\frac{1}{f=50}$ Sec.

### 2.2. Reconstruction and signal-piloted acquisition

The considered instances of power signals are reconstructed. It allows evaluating the SPADC [35]. Up-sampling is realized by using a combination four cascaded cubic-spline interpolators [36]. It transforms the incoming signal *y*(*t*_*n*_) in its quasi analog version $\tilde{y}(t)$. The relationship between $\tilde{y}(t)$ and *y*(*t*_*n*_) is given by Eq (5). Onward, the noise $\tilde{n}(t)$ is added in $\tilde{y}(t)$ to obtain the noisy signal, given by Eq (6). The probability density function of $\tilde{n}(t)$ is given by Eq (7). Where, *μ* is mean and σ is the standard deviation.

$$\tilde{y}(t)=y(t_{\frac{n}{U}}).$$ (5)

$$\tilde{x}(t)=\tilde{y}(t)+\tilde{n}(t).$$ (6)

$$\text{p}(\tilde{x})=\frac{1}{\sigma \sqrt{2\pi }}{\text{e}}^{\frac{-{\left(\tilde{x}-\mu \right)}^{2}}{2\sigma^{2}}}.$$ (7)

The contemporary automated PQDs identifiers are frequently using the ADCs. The acquisition of signals is typically founded on the time-invariant and fix-rate concept [36]. The implementation of these systems is thus realized for the extreme scenarios [36]. However, such solutions are not efficient in the case of unpredictable PQDs [37]. To compensate these limitations, the SPADCs are used. Based on the Level-Crossing Sensing (LCS), a real-time self-organization capability is available in SPADCs [37]. It permits to change their rate of acquisition as per changes in the incoming signal, $\tilde{x}(t)$[37]. The process of sampling is triggered by the predefined thresholds crossings, made by $\tilde{x}(t)$. Consequently, samples are non-uniformly arranged in time. The process is given by Eq (8). Where *dt*_*n*_ is the time elapse among the current instant of sampling, *t*_*n*_, and the prior one, *t*_*n*-1_.

$$t_{n}=t_{n-1}+dt_{n}.$$ (8)

For SPADC, the amplitude of a sample is equivalent to one of the preconfigured thresholds and the instant of that sample is recorded with the help of an appropriate timer circuit [35]. A 4-Bit resolution uniform quantization based SPADC is used for acquiring the power signals. The choice of resolution is made while achieving a favourable compromise among the system implementation and computational complexities and precision in the detection of PQDs.

In contrast to conventional ADCs, the signal-to-noise-ratio (SNR) of SPADCs is independent of the quantizer resolution. It solely depends on the operating frequency, *F*_*Timer*_, of the timer circuit and is given by Eq (9). Where, *T*_*timer*_ is the timer step and is equal to $\frac{1}{F_{Timer}}$ [35]. *f*_*sig*_ = 50Hz is the desired frequency of power supply. In this study, *F*_*Timer*_ = 1MHz is selected. It renders an ideal SPADC SNR of more than 12-Bit, which is a suitable resolution for the automated PQDs recognition methods [15, 16, 19–23].

$$SNR_{dB}=-11.19-20log(f_{sig}.T_{timer}).$$ (9)

### 2.3. Adaptive-rate segmentation

**Fig 2:**
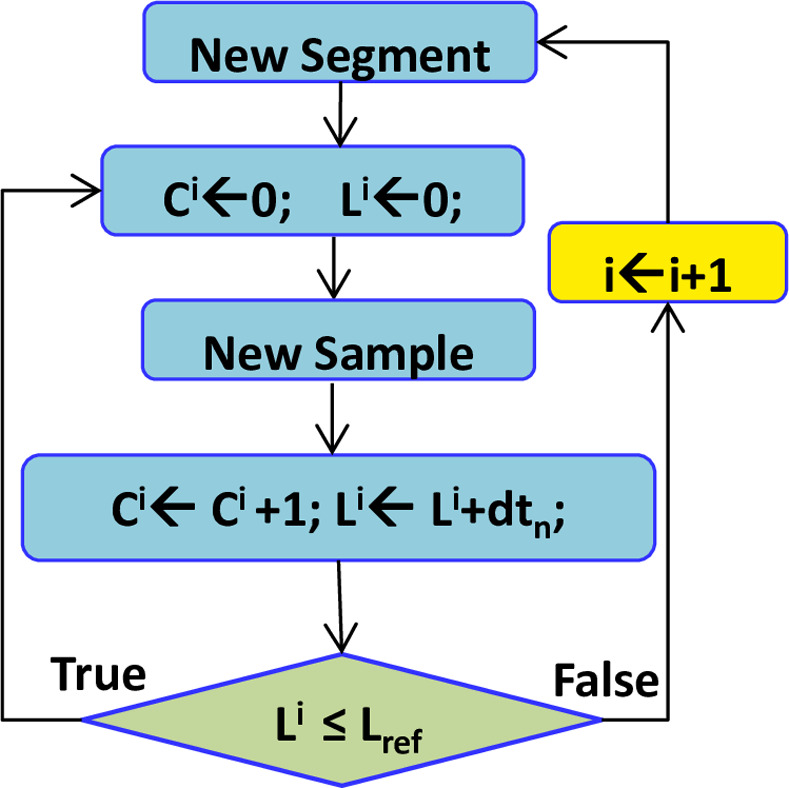
Flow chart of the MASA.

To efficiently segment the SPADC outcome, a modified version of the Activity Selection Algorithm is used [35, 37]. It is named as MASA and is realized by the use of implicit information, available in the employed sampling process non-uniformity. It permits to segment the SPADC output in a real-time and self organised manner. The functioning of MASA is depicted with the help of a flow chart, shown in Fig 2.

In Fig 2, *C*^*i*^ is the count of samples and *L*^*i*^ is the length in seconds of the *i*^*th*^ selected segment *W*^*i*^. In the beginning, *C*^*i*^ and *L*^*i*^ are reset to zero. Onward, keep adding samples in *W*^*i*^. For each added sample, *x*_*n*_, the *C*^*i*^ and *L*^*i*^ are updated and are respectively given by Eqs (10) and (11). The process continues unless *L*^*i*^ ≤ *L*_*ref*_. Once, the condition *L*^*i*^ ≤ *L*_*ref*_ violates, *i* is incremented and the processing of new segment begins. In this case, *L*_*ref*_ = 100-mSec is the selected upper bound on the length of segments [35]. This choice of *L*_*ref*_ is made in an incremental manner while taking in account the statistical characteristics of the intended PQ events [33, 34]. This decision is made as a function of attaining the best compromise among the system performance and processing complexity. Consequently, for this choice of *L*_*ref*_, for each instance of 0.5-Second duration, the MASA delivers 5 selected segments. Due to the adaptive-rate and signal-piloted acquisition approach, the samples count for each selected segment could vary. It adapts in accordance with the $\tilde{x}(t)$ variations.

$$C^{i}=C^{i-1}+1.$$ (10)

$$L^{i}=L^{i-1}+dt_{n}.$$ (11)

### 2.4. Features extraction

The Thanks to the SPADC and the ASA processes, the time domain data possesses significant information about the signal frequency content [35]. Thus, compared to alternative methods which are based on frequency or time-frequency representation, the designed method does not require any computationally complex transformation [15–17, 21–24, 26]. In the time-domain, the signal-piloted acquisition based data is immediately used to mine the relevant classifiable features.

**Fig 3:**
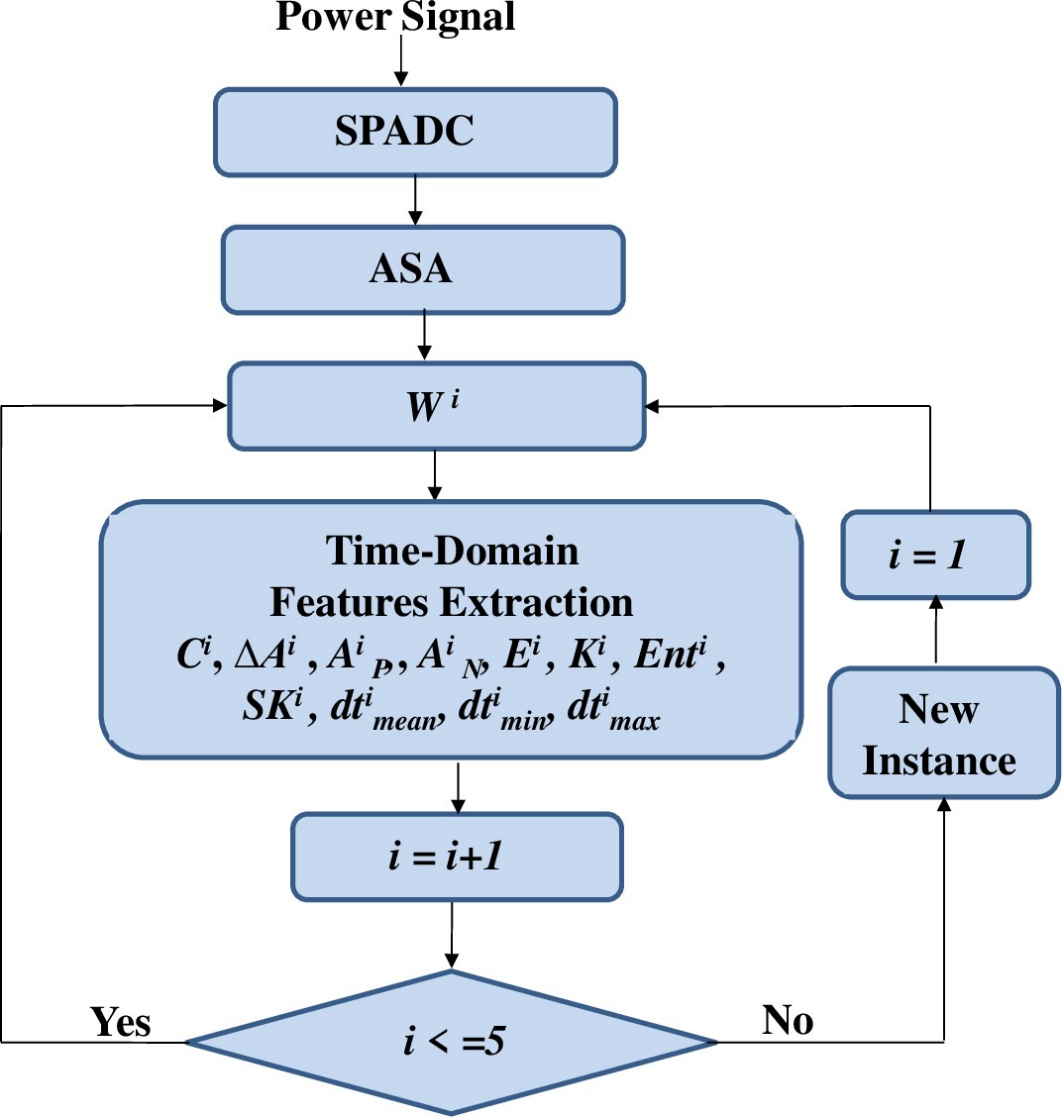
The features extraction principle.

The ASA delivers 5 selected segments per instance. Eleven different features, in time-domain, are mined for each segment. In this way 55 features are used to present each instance. The mechanism is also shown in Fig 3. It presents that for *W*^*i*^ the mined attributes are: the count of samples, *C*^*i*^, the positive peak, $A_{P}^{i}$, the energy, *E*^*i*^, the negative peak, $A_{N}^{i}$, the peak-to-peak amplitude, Δ*A*^*i*^, the skewness *SK*^*i*^, the average sampling step, $dt_{mean}^{i}$, the entropy, *Ent*^*i*^, the minimum sampling step, $dt_{min}^{i}$, the kurtosis, *K*^*i*^ and the maximum sampling step, $dt_{\max}^{i}$.

### 2.5. Classification methods

After features extraction each intended instance is represented by 55 features. The employed dataset has four power signals classes. 800 instances are considered for all classes, resulting in a selected features dataset, *P*, with a dimension of 800x55. The well-known robust methods, k-Nearest Neighbor (KNN), Support Vector Machine (SVM), ANN and Naïve Bias (NB), are used for classification. Because of the small data collection, the classification’s accuracy could be biased. The cross-validation scheme is frequently used to compensate this impact of biasness and to avoid over fitting [38]. Accordingly, this analysis uses 10-fold cross-validation. The MATLAB program is used to carry out the classification [32].

#### 2.5.1. k-Nearest Neighbor (KNN)

The KNN is regarded as computationally effective but powerful classifier [38]. It employs features’ distance to label the test samples. A sample, *z*, is classified by majority voting of its *K* neighbours, determine the maximum probability [38]. The process is given by Eq (12). Where, *C* is the features set of total number of classes and *y* is the output label.

$$p(y=j|C=z)=\frac{1}{K}\sum_{i\in A}I(y^{i}=j)$$ (12)

The Euclidian distance measure is used. The choice of *K* should be appropriately made. In a step-by-step fashion, various *K* values are evaluated and the value with the least classification error is chosen. The best results are obtained for *K* = 5 in this study.

#### 2.5.2. Naïve Bayes (NB)

One of the most common machine-learning techniques is the Naïve Bayes classifier. Its "naive" presumption sets the conditions for all characteristics to be independent. Parameters are separately studied with each attribute and this greatly simplifies the learning. Consequently, because of its independence presumption that simplifies the algorithm [39], the Naïve Bayes algorithm is often used for classification of a high-dimensionality features vector. It predicts its own probability for each class when classifying a sample [38]. The process is given by Eq (13). Where *z* represent a sample under test and *y* is the predicted label. *C* is the features set of total number of classes. *p* is the probability and *argmax* outcomes the maximum value for the targeted function.

$$y=argmax_{z}[p(z|C)*\prod_{i=1}^{n}p(y^{i}|z)]$$ (13)

In this study, the Gaussian Naïve Bayes classifier is used with a batch size of 100.

#### 2.5.3. Support Vector Machine (SVM)

The support vector machine locates a hyperplane in an N- spacer that classifies the test samples distinctly [40]. The aim is to find a plane with the greatest range. Maximizing the gap from the margins gives sufficient clarification. Therefore, potential test samples can be identified with better assurance [40]. Eq (14), mathematically described the process of separating hyperplane. Where, ***X*** is the sample vector ***X*** = [*x*_1_, *x*_2_…*x*_*q*_] having *q* attributes, ***W*** = [*w*_1_,*w*_2_…*w*_*q*_] is the weights vector, and *b* is a scalar bias. SVM is particularly good for solving two class problems. Various methods can be employed while dealing with multiple classes. In this case, the One-vs-All strategy is used with Sequential Minimal Optimization (SMO) to train the classifier. In a step-by-step manner, the best suitable value of the regularisation parameter is found to be 100 while using the polynomial kernel.

$$h(X)=sign(W\cdot X^{T}+b)$$ (14)

#### 2.5.4. Artificial Neural Network (ANN)

The ANN follows the principle of biological neural networks [41]. The fundamental building block of an ANN is an artificial neuron. Multiplication, summation, and activation are the three fundamental stages of an ANN. It is a network of the input and output units, interlinked via hidden layers. The weight of each interlinking node is tuned during the training process. It permits the development of non-linear and complicated input-output relations. Onward, this knowledge is used for labelling the test data. The major drawback of an ANN is the unknown behaviour of neural networks that may result in unexplainable classification output. Furthermore, as the number of hidden layers grows, ANN necessitates a significant volume of data for proper training. The principle of labelling a sample *z* as *y* is given by Eq (15). Where *f* is a nonlinear activation function. ***X*** is the features vector ***X*** = [*x*_1_, *x*_2_…*x*_*q*_] having *q* attributes and ***W*** = [*w*_1_,*w*_2_…*w*_*q*_] is the weights vector. *b* is a scalar bias.

y=f(X⋅W+b) (15)

The multilayer perceptron (MLP) technique is used in this study. The best precision is secured while using three hidden layers, each containing 50 nodes for the reduced setting and 90 nodes for the full setting.

### 2.6. Performance measures

#### 2.6.1. The samples ratio (*SR*)

It presents the ratio between the counts of power signal samples, collected in the traditional case and the proposed case for a given time length. Let *SR*^*i*^ be the samples ratio for *W*^*i*^ then it is given by Eq (16). Where, *N*_*r*_ and *C*^*i*^ are respectively the number of samples exist in *W*^*i*^ for the classical and the devised approaches.

$$SR^{i}=\frac{N_{r}}{C^{i}}.$$ (16)

#### 2.6.2. The compression ratio (*R*_*COMP*_)

This tests the success of suggested method in reducing the amount of information to be transmitted to the classifier. The comparison is made with traditional methods which, without any reduction in the dimension, conveys the captured power signals to the classifier [2]. In the conventional and proposed cases, let *N*_*r*_ and *P* present datasets that must be transmitted and classified, respectively. In this case, each element of *N*_*r*_ and *P* is presented with 12-Bit resolution [2, 33, 34]. Therefore, the compression ratio in bits, *R*_*COMP*_, can be computed by using Eq (17).

$$R_{COMP}=\frac{N_{r}}{P}.$$ (17)

#### 2.6.3. The classification accuracy (Acc)

The proposed solution is beneficial because of its signal-piloted nature. It can bring real-time reduction in the count of samples, compression and processing effectiveness [37]. However, its classification performance can be compromised compared to the traditional fixed-rate equals.

The accuracy score tests the success of suggested method in categorizing the PQDs. It is the percentage of correctly identified labels. The process is given by Eq (18). Where, true positive, true negative, false positive and false negative are respectively denoted by TP, TN, FP and FN. In the studied case it provides an appropriate evaluation of the classification performance because all the considered class sizes are equal.

$$Acc=\frac{\text{TP}+\text{TN}}{\text{TP}+\text{TN}+\text{FP}+\text{FN}}\times 100.$$ (18)

#### 2.6.4. The F-measure (F1)

The *F1*seeks to find a balance between values of recall and precision. In the analyzed scenario, all groups have the same data size, so we simply use the F-measure macro given by Eq (19), where *precision* = $\frac{TP}{\left(TP+FP\right)}$ and *recall* = $\frac{TP}{\left(TP+FP\right)}$.

$$F=\frac{2*precision*recall}{precision+recall}.$$ (19)

#### 2.6.5. The area under the ROC curve (AUC)

The ROC curves, on the horizontal axis, map the *TN* rate against the *TP* rate on the vertical axis. The ROC curve is closely based on the specifics of the particular test data set. This dependency can be reduced by using the cross-validation technique. It supposes that the probability estimates of the predictors which are constructed from the various training sets are all built on random data samples of the same size. The larger the area underneath the ROC curves, the better is the classifier, and vice versa [42].

#### 2.6.6. The kappa index

It statistically measures the agreement between two clustering. The Cohen’s kappa measure is used. It is popularly employed for evaluating clustering results and is expressed by using Eq (20). Where, the likelihood of agreement between the expected and target labels is denoted by *po*, while *p*_*e*_ is the probability of such an agreement happening by chance.

$$kappa=1-\frac{1-p_{0}}{1-p_{e}}.$$ (20)

## 3. Results

**Fig 4:**
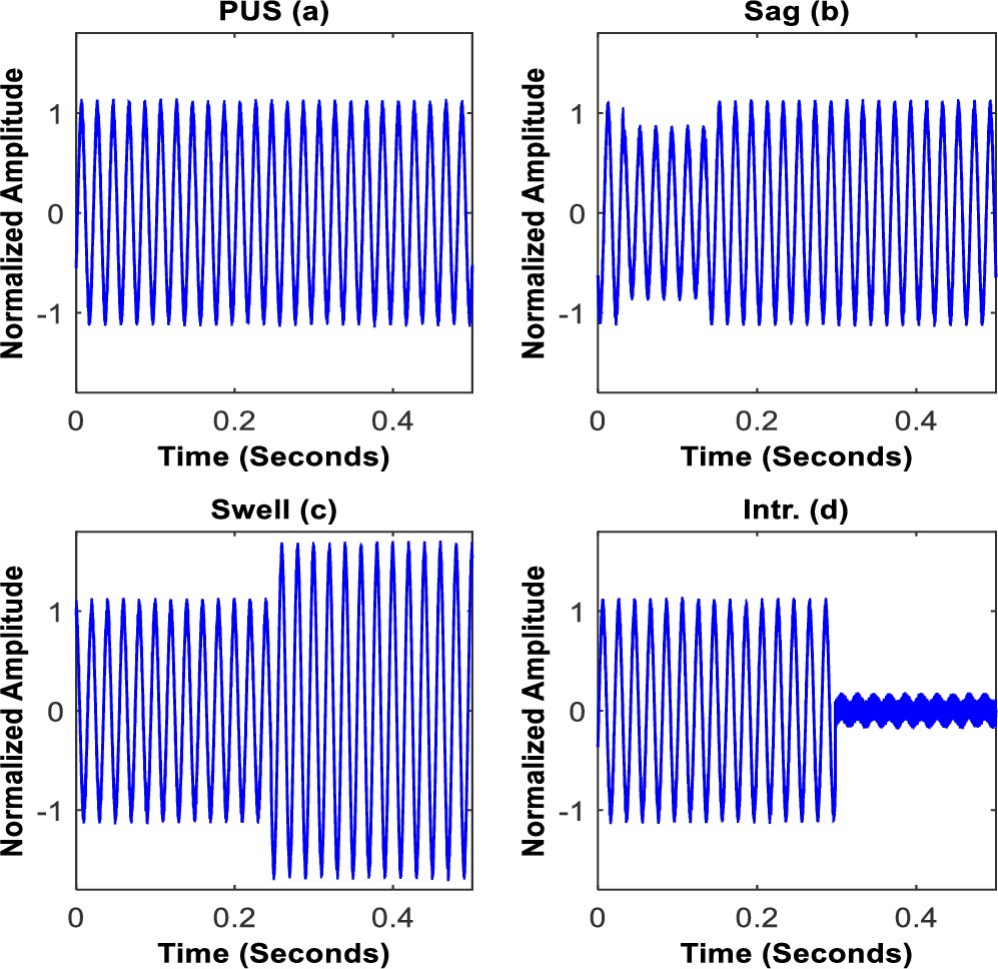
The instances for pure signal, sag, swell and intr.

The In this study four major categories of power signals are considered namely PUS, sag, swell and intr. Fig 4 displays examples of the normalized waveforms of the intended power signals.

**Fig 5:**
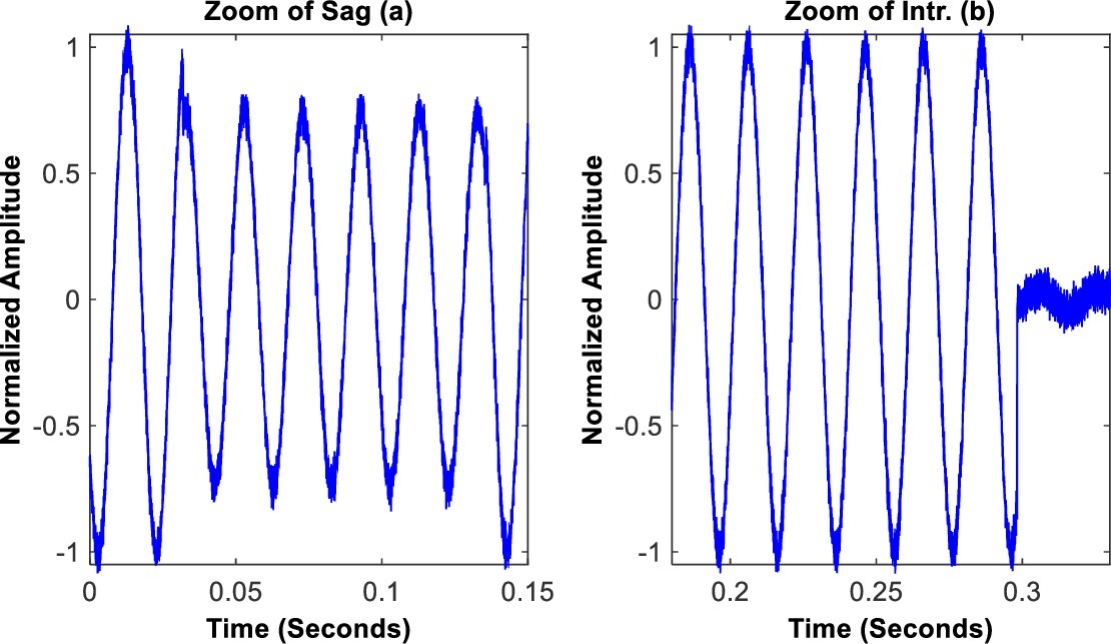
Zooms of instances of sag and intr.

In order to show the impact of noise, the zoom of waveforms for the case of typical sag and intr. Instances are respectively shown in Fig 5A and 5B.

**Fig 6:**
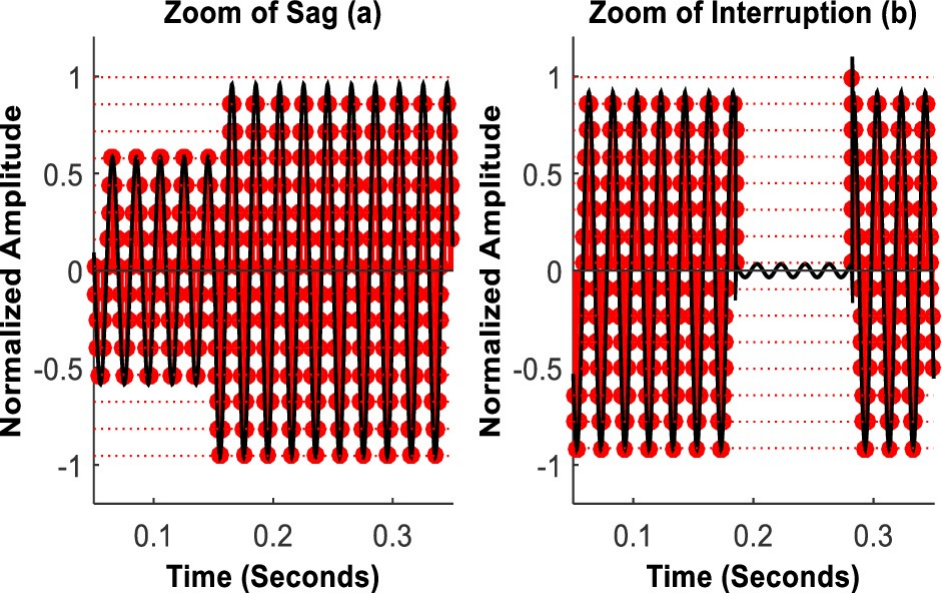
The instances acquired with a 4–bit resolution SPADC for sag (left) and intr. (right).

The considered power signal instances are generated at a sampling rate of 6.4 kHz. Each instance subsequently undergoes through the up-sampling by a factor of 100. Examples of zooms of typical instances, digitized with a 4-bit resolution SPADC, of sag and intr. are shown in Fig 6.

Fig 6 indicates gains of using SPADC. It supports that it updates its sampling frequency, for a given resolution, based on variations in the incoming signal [35].

**Fig 7:**
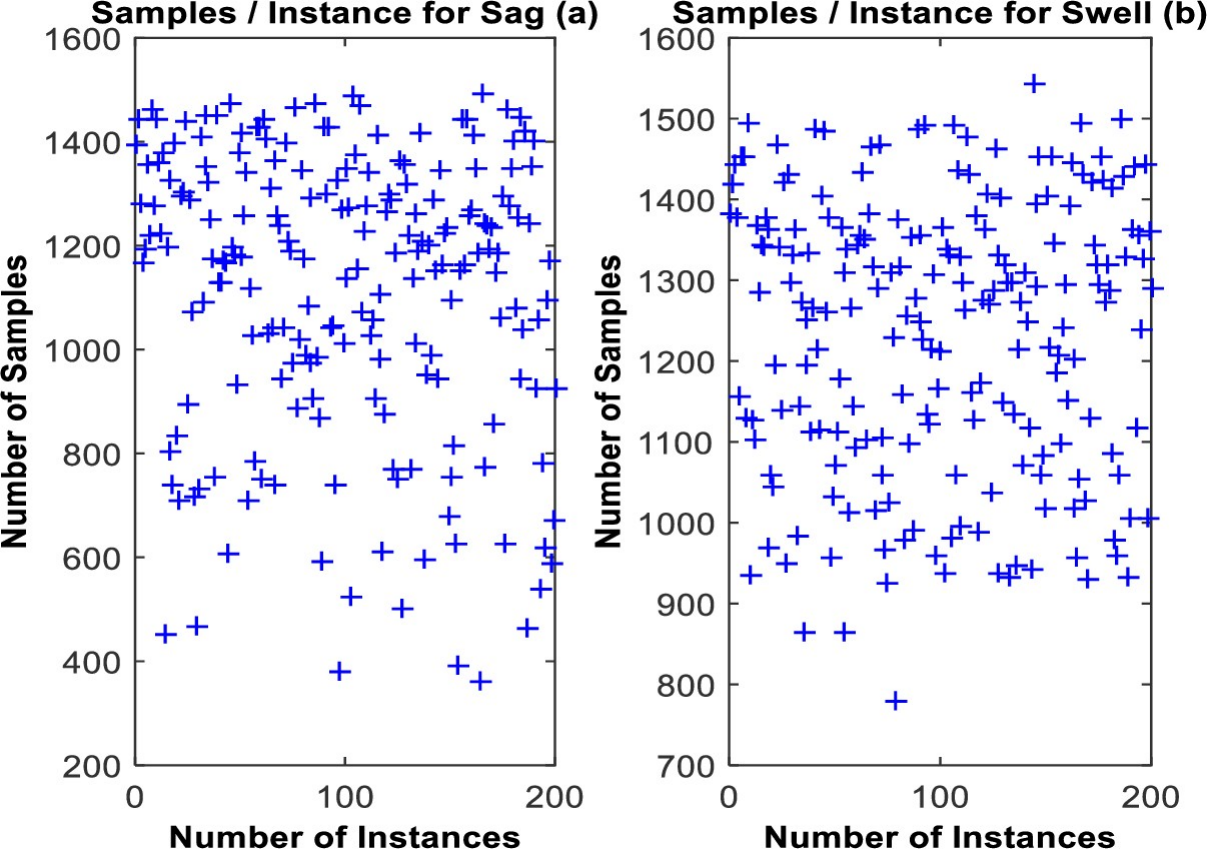
Number of samples per instance for sag (left) and swell (right).

In this study, the ASA is used to segment the output of SPADC [35]. For each incoming power instance, 5 selected segments are delivered by the ASA. However, the count of thresholds, *C*^*i*^, changes according to the time variations in the pattern of incoming instance. The phenomenon is clear from Fig 7, which shows the total number of samples, collected for each considered instance of sag and swell. It also confirms the random nature of studied dataset.

Conventionally, $\tilde{x}(t)$ is continuously acquired at 6.4 kHz regardless of its variations. Therefore, an augmented collection and processing of redundant samples happen. 640 samples per segment are collected for *L*_*ref*_ = 100mSec. Consequently, the post-processing modules analyse redundant data which results in an increased processing load, transmission and power consumption overhead.

The overall sample ratios, for 200 instances of each class, are calculated by using Eq (7). Findings are outlined in Table 1. This reveals that the designed approach reaches an overall average samples count reduction, obtained for all intended classes, of 6.75-fold.

**Table 1:** Summary of the samples ratios.

Power Signal	Maximum	Minimum	Mean	Median
Sag	17.68	4.28	6.29	5.41
Swell	8.22	4.15	5.30	5.03
PUS	4.27	4.26	4.26	4.26
Intr.	71.11	4.31	11.14	7.97

Each intended power instance is presented by 55 features, after application of the proposed features extraction technique. On the other hand, each power instance is originally composed of 3200 samples. It results in a *R*_*COMP*_ = 58.18. This promises the same reduction factor in data transmission operation, bandwidth usage, and power consumption plus classifier’s computational efficiency.

In total 800 instances are studied, for the considered categories of power signals, in this study. To account for the size limit of the dataset, the 10-fold cross-validation approach is used [42]. As mentioned in Section 2.5, the parameters of each classifier are configured during the training process. The evaluation measures namely Acc, F1, AUC and Kappa are measured while classifying the considered dataset.

The obtained percentage accuracies of identification are listed in Table 2 respectively for the KNN, NB, SVM and ANN classifiers.

**Table 2:** Accuracy scores for power signals recognition, obtained by different classifiers.

Power Signal	Acc (%age)			
	KNN	NB	SVM	ANN
Sag	91.55	92.46	96.25	94.62
Swell	99.05	98.13	99.87	100
PUS	97.71	100	99.48	99.34
Intr.	92.72	93.16	96.61	94.97

Table 2 outlines that for KNN classifier the accuracy score of the classification of PQ signals received is 91.55% for the sag, 99.05% for the swell, 97.71% for the pure signal and 97.72% for the interruption. The overall average classification accuracy score of KNN is 95.26%.

The accuracy score for NB classifier is 92.46% for the sag, 98.13% for the swell, 100% for the pure signal and 93.16% for the interruption. For all categories, the overall classification accuracy score of NB is 95.94%.

The accuracy score of SVM classifier is 96.25% for the sag, 99.87% for the swell, 99.48% for the pure signal and 96.61% for the interruption. For all categories, the overall classification accuracy score of SVM is 98.05%.

The accuracy score of ANN classifier is 94.62% for the sag, 100% for the swell, 99.34% for the pure signal and 94.97% for the interruption. For all categories, the overall classification accuracy score is ANN is 97.23%.

The obtained average F1, AUC and Kappa values for each considered classifier are listed in Table 3. It reveals that, as compared to KNN and NB, the suggested signal-piloted processing chain in combination with SVM and ANN identifies better the intended PQDs. It is because the SVM and ANN are less likely to be biased. However, KNN and NB can be easily distracted particularly if the data contains outliers. This statement is further enforced by the findings, average scores across F1, AUC and Kappa, which are summarized in Table 3.

**Table 3:** Performance of considered classifiers using F1, AUC and Kappa metrics on the test dataset.

Classifier	F1	AUC	Kappa
KNN	0.910	0.970	0.880
NB	0.920	0.978	0.893
SVM	0.961	0.981	0.948
ANN	0.945	0.991	0.927

We considered the detailed classification outcomes for each expected class for further study, as shown in Tables 4 and 5. Relative to KNN, NB and ANN, the total FP and FN counts using SVM are the lowest, thus confirms the better performance of the SVM compared to other algorithms under consideration.

**Table 4:** Confusion matrices for the KNN and the NB classifiers.

KNN& NB		Predicted (KNN)				Predicted (NB)			
		Sag	Swell	PUS	Intr.	Sag	Swell	PUS	Intr.
Actual	Sag	168	0	10	22	185	4	0	11
	Swell	2	193	5	0	6	193	0	1
	PUS	0	0	200	0	0	0	200	0
	Intr.	33	0	2	165	39	3	0	158

**Table 5:** Confusion matrices for the SVM and the ANN classifiers.

SVM & ANN		Predicted (SVM)				Predicted (ANN)			
		Sag	Swell	PUS	Intr.	Sag	Swell	PUS	Intr.
Actual	Sag	184	0	3	13	179	0	4	17
	Swell	1	199	0	0	0	200	0	0
	PUS	0	0	200	0	0	0	200	0
	Intr.	13	0	1	186	22	0	1	177

## 4. Discussion

The designed PQDs recognition mechanism is evaluated by using the real-like model-based power signal instances. The PQ events are randomly generated as per the IEEE-1159 standards [2, 33, 34]. The obtained results are appealing and are outlined in Section 3. The technique yields an overall 16.87-fold reduction in count of collected samples over traditional equals. This assures a major decrease in arithmetic complexity and the overhead capacity consumption of the proposed solution. In addition, SPADC embedding and features selection functionality in PQDs elucidators greatly decreases data storage and communication operation between frontend sensors and cloud-based classifier relative to traditional methods. The compression ratio achieved is 58.18-fold for the studied case. Moreover, features are extracted in time-domain without the application of any computationally complex transformation. This confirms the superiority of the suggested solution over the existing ones [9, 16, 23, 43]. The used SPADC is of much inferior, 4-Bit, resolution. Nonetheless, a 12-Bit ADC is used in the fix-rate equals to acquire the power signals [15, 16, 19–23]. This guarantees a significant decrease in the processing cost and complexity of the system’s hardware relative to conventional equivalents.

In the fix-rate solutions, a lower sampling rate and a bigger quantization step can also be used. However, it decreases the ADC SNR [35] and can lower the precision of automated PQDs classification. The SNR of SPADC, does not depend on the quantum value and is based on the timer circuit frequency which is used to measure the sampling instants [35]. Furthermore, the uniform sampling-based approach would lack in extracting the necessary information of the PQD instances in solely time-domain. Therefore, these techniques use analysis methods in both time and frequency domains for mining the classifiable attributes. It can increase the complexity of computing and latency relative to the suggested method.

The accuracy score of 98.05% is secured with the SVM classifier for the studied scenario. The ANN follows with 97.23% accuracy. The NB attains the accuracy of 95.94% and the KNN achieves the lowest average accuracy of 95.26%. For the studied case, the SVM performs better as compared to the ANN, NB and KNN. It is also confirmed by other evaluation scores such as F1, AUC and Kappa statistics. It is because of the SVM ability to successfully prune the most unnecessary possibilities when classifying an instant under test. This states that the suggested framework, based on the SPADC, ASA, time-domain features mining and the SVM offers the best interpretation of the intended power signals.

The idea of integrating the signal-piloted acquisition and adaptive-rate time-domain features mining in the PQDs elucidators is new. Comparing the suggested method with the state-of-the-art approaches is not obvious, as they are based on standard fixed-rate sampling and processing [9, 15, 16, 20–23]. In addition, each research uses various categories of the PQDs, processing and analysis tools and classification algorithms. However, a comparison is made between the key preceding studies, carried out on synthetic PQDs. For all considered studies, the best classification accuracies are outlined in Table 6. It reveals that the accuracy of PQDs categorisation, attained by the suggested method, is similar or superior than the fix-rate equivalents.

**Table 6:** Comparison with state-of-the-art methods.

Study	Features Extraction	Classification	Accuracy (%)
[19]	Tim-domain and frequency-domain statistical features.	Artificial Neural Network (ANN)	96.03
[15]	singular spectrum analysis and Curvelet transform	Deep convolutional neural networks	100
[20]	Higher-order Statistical features	Higher-Order Statistical Estimator	83.00
[16]	Sparse signal decomposition with hybrid dictionary	Hierarchical Decision Tree Algorithm	97.3
[21]	discrete wavelet transform and entropy norm	ANN	99.66
[22]	Non-dominated sorting Genetic algorithm based on S-transform and time–time transform	Decision tree	99.93
[23]	Independent component analysis	Multi Layer Perceptron (MLP)	97.00
This Study	SPADC with ASA and time-domain statistical features	SVM	98.05

## 5. Conclusion

A novel approach of automatically identifying the primary voltage and transient-based power quality events is proposed. It is developed by intelligently combining the signal-piloted acquisition, adaptive-rate segmentation, time-domain features extraction, and machine learning tools. In contrast to the counterparts, this method does not employ arithmetically complex transformations for feature extraction. It is demonstrated that signal-piloted sampling has resulted in a 16.87-fold decrease in the amount of recorded information compared to the fix-rate counterparts. It aptitudes an enhanced performance in terms of processing and power consumption diminishing. Additionally, a similar reduction is promised in the information management activity and post-classification complexity. The developed framework also offers a simplified hardware complexity while achieving an equivalent or superior PQDs recognition accuracy score with respect to the contemporary equals. The designed solution attains the best average classification accuracy score of 98.05%. It confirms the benefit of incorporating the suggested method in modern PQDs management solutions.

In this study a uniform quantization based SPADC is investigated while considering four major classes of the power quality disturbances. Investigating the application of non-uniform quantization-based SPADC with broader categories of power quality disturbances is future work. Another prospect is to evaluate the possible integration of other robust classifiers such as Rotation Forest, Random Forest, ensemble algorithms, and deep learning methods in the suggested solution.

## Supporting information

S1 Dataset (ZIP)

S1 File (DOCX)
