# Supplementary material for: Signal-piloted processing and machine learning based efficient power quality disturbances recognition
Source: PLoS One. 2021 May 28;16(5):e0252104. doi: 10.1371/journal.pone.0252104 (PMC8162588; doi:10.1371/journal.pone.0252104)
Supplement: S1 File — (DOCX) [file pone.0252104.s002.docx]

Signal-piloted processing and machine learning based efficient power quality disturbances recognition

**Highlights:**

This paper suggests an efficient and precise solution for major power quality voltage and transient disturbances recognition, without any computationally complex transformation based approach of features extraction, by directly processing the PQ signals waveforms in time-domain.

It is realized by:

- Intelligently combining the signal piloted Analog to Digital Converters (SPADCs), Activity Selection Algorithm (ASA), time-domain features extraction and machine learning algorithms for an efficient classification of PQ events:

1. The SPADCs acquire the PQ signals at adaptive rates,
2. The ASA is used for effective segmentation of SPADC outcome,
3. Each selected segment is analyzed directly in time-domain by a features extractor to mine its classifiable attributes,
4. The performance of k-Nearest Neighbor, Naïve Bias, Artificial Neural Network and Support Vector Machine is studied for the recognition of PQ events by using the forehand extracted features,
5. To avoid over fitting and any biasness, the classification performance is evaluated by using the 10-fold cross validation and the multiple measures such as accuracy, F-measure, area under the ROC curve (AUC) and Kappa statistics.
6. The suggested method secures a 16.87-fold decrease in the amount of acquired information relative to conventional equals while attaining the classification accuracy score of 98.05%.
